# The prevalence of underweight, overweight and obesity in children and adolescents from Ukraine

**DOI:** 10.1038/s41598-018-21773-4

**Published:** 2018-02-26

**Authors:** Katarzyna Dereń, Serhiy Nyankovskyy, Olena Nyankovska, Edyta Łuszczki, Justyna Wyszyńska, Marek Sobolewski, Artur Mazur

**Affiliations:** 1 Medical Faculty, University of Rzeszów, Rzeszów, Poland; 2 Pediatrics Department, Danylo Halytsky L’viv National Medical University, L’viv, Ukraine; 3 Department of Pediatrics and Neonatology, Danylo Halytsky L’viv National Medical University, L’viv, Ukraine; 4 Faculty of Management, Rzeszów University of Technology, Rzeszów, Poland

## Abstract

The aim of this study was to evaluate the prevalence of overweight, obesity and underweight in children from Ukraine. A cross-sectional study was performed on data collected from a representative sample of Ukraine children (13,739 children (boys 48%, girls 52%) aged 6.0–18.9 years). The measurement of body weight was performed with medical scales and height was measured using a stadiometer. Based on the results obtained, body mass index (BMI) was calculated. Three criteria were used to define childhood underweight, overweight and obesity: The International Obesity Task Force (IOTF) reference, World Health Organization (WHO) child growth standard and The US Center for Disease Control and Prevention (CDC). The combined prevalence of obesity and overweight among children aged 6–18 years old was 12.1%, 17.6%, and 12.6% based on the IOTF reference, WHO growth standard, and the CDC, respectively. Obesity was 2.1%, 4.2%, and 3.6% respectively. Significantly more girls were underweight than boys. Furthermore, a higher prevalence of overweight and obesity was diagnosed in younger that older Ukrainian population.

## Introduction

The negative impact of being underweight, overweight, or obese on the health and development of children and adolescents can also extend into adulthood, increasing the risk of chronic non-communicable diseases and disability. An enormous epidemic of lifestyle diseases, including obesity, are now among the biggest problems of modern medicine. In the development of obesity, particular attention is directed to the periods of pre-school and adolescence regarded as times of risk for the development and maintenance of obesity that can lead to consequences in adulthood^1^.

The problem of low or excessive body weight concerns countries with different levels of socio-economic development. This is a medical, social, and economic issue. The World Health Organization (WHO) has voiced concerns that there are more than 110 million children with excessive body weight worldwide^2^. In addition, many sources report that the problem of underweight individuals affects poor and developing countries and the prevalence of undernourishment may be caused by, for instance, an insufficient amount of funds to buy food^3^.

Knai *et al*. reported high levels of overweight and obesity with an increasing tendency among the population of adults and children, not only in Western Europe, but also in Eastern Europe^4^. The data available in the literature rarely address the subject within countries of the former Soviet Union. One such country is Ukraine, a developing country located in Eastern Europe with a population of over 42 million^5^. Unfortunately, it has also been affected by war for the past several years.

Available WHO data reports that 53.5% of adults are overweight and 21.3% are obese^6^. The percentages in the population under 20 years of age is similar to that for adults. In terms of the prevalence of overweight and obesity in adolescents aged 10–19 years, 22% of boys and 12% of girls aged 11 are overweight. Among 13-year-olds, the figures are 21% for boys and 9% for girls and in 15-year-olds it is 17% and 8% respectively^7^. According to WHO, obesity among children under five years of age amounts to 26%^8,9^.

There is even less data available in the literature on the prevalence of underweight and malnutrition among children and youth in Ukraine^9^. Due to the lack of up to date data in the literature, research on the issues of malnutrition, overweight and obesity in children in Ukraine deserves attention.

The aim of this study was to evaluate the prevalence of overweight, obesity and underweight in children from Ukraine.

## Results

The characteristics of the number and percentage of children in different age groups depending on sex is presented in Table 1. The most numerous group was represented by children aged 12 and 14 years (10.1 and 10.8% respectively), the groups of 8–11, 13 and 15 year-olds were also numerous. The least numerous groups were those of 18 (0.5%), 6 (3.2%) and 17 (4.1%) years, respectively. The mean age of the participants was 11.5 years. The average age of girls was 11.43 years and boys 11.56 years. In the allocation of children to age groups, the number of days elapsed since their last birthday was taken into account (i.e. 6-year-olds: 6.00–6.99 years, 7-year-olds: 7.00–7.99 years, and so forth).

**Table 1:** Absolute frequencies and percentages of children disaggregated by age and sex.

Age [yrs]	Boys n (%)	Girls n (%)	Total n (%)
6	214 (48.2)	230 (51.8)	444 (3.2)
7	601 (51.3)	571 (48.7)	1172 (8.5)
8	615 (47.5)	681 (52.5)	1296 (9.4)
9	650 (49.4)	666 (50.6)	1316 (9.6)
10	638 (49.4)	654 (50.6)	1292 (9.4)
11	604 (47.6)	666 (52.4)	1270 (9.2)
12	675 (48.7)	710 (51.3)	1385 (10.1)
13	624 (46.5)	717 (53.5)	1341 (9.8)
14	707 (47.6)	778 (52.4)	1485 (10.8)
15	587 (46.5)	675 (53.5)	1262 (9.2)
16	395 (46.6)	452 (53.4)	847 (6.2)
17	257 (45.6)	306 (54.4)	563 (4.1)
18	29 (43.9)	37 (56.1)	66 (0.5)
Total	6596 (48)	7143 (52)	13739 (100)

Classification of children according to BMI for different reference values gives different results. The percentage of obese children ranges from 2.1% for the IOTF classification, 3.6% for the CDC to 4.2% for the WHO. Large differences exist in the assessment of underweight. The IOTF classification, for which the BMI was 18 for an adult, led to a significant percentage of children being classified as underweight. But it is crucial that some trends - more obese among boys and more obese among younger children remain the same regardless of how the BMI is classified (Table 2).

**Table 2:** Prevalence (%) of underweight, overweight or obesity among Ukraine children and adolescents based on three international BMI references.

Age group (years)	Underweight %			Normal %			Overweight %			Obesity %		
	IOTF	WHO	CDC	IOTF	WHO	CDC	IOTF	WHO	CDC	IOTF	WHO	CDC
All												
6–12	14.7	5.2	9.2	69.9	71.7	74.0	12.3	16.8	11.4	3.1	6.3	5.4
13–18	15.9	4.4	7.8	77.0	86.0	85.7	6.6	8.5	5.4	0.5	1.0	1.0
6–18	15.2	4.9	8.6	72.8	77.5	78.8	10.0	13.4	9.0	2.1	4.2	3.6
Boys												
6–12	11.0	4.5	7.7	72.1	67.8	72.2	13.5	19.2	13.2	3.4	8.6	6.9
13–18	10.4	3.9	7.0	80.4	83.5	84.7	8.6	11.1	6.8	0.7	1.5	1.5
6–18	10.8	4.2	7.4	75.4	74.0	77.1	11.5	16.0	10.7	2.3	5.8	4.8
Girls												
6–12	18.2	5.9	10.6	67.8	75.4	75.8	11.2	14.5	9.8	2.8	4.2	3.9
13–18	20.7	4.9	8.5	74.0	88.3	86.7	4.8	6.3	4.2	0.4	0.6	0.6
6–18	19.3	5.5	9.7	70.4	80.8	80.3	8.5	11.1	7.4	1.8	2.7	2.5

CDC - The US Center for Disease Control and Prevention, IOTF - The International Obesity Task Force reference, WHO - World Health Organization child growth standard.

Differences were observed in the distribution of the BMI category based on the IOTF reference among girls and boys (Table 3). Significantly more girls (1,377) were underweight than boys (711) (p < 0.001). Normal body weight was found in 75.4% of boys and 70.4% girls, while the percentage of overweight and obese was found to be 13.9% for boys and 10.3% for girls. Boys are, therefore, more likely to be overweight, and girls more prone to be underweight. Using the concept of the prevalence ratio and logistic regression models it was found that overweight and obesity were less common among girls (prevalence ratio 0.747). Less girls had normal body weight than boys (70.4% vs. 75.4%), but significantly more (19.3% vs. 10.8%) were underweight (p < 0.001).

**Table 3:** Prevalence ratio for underweight, overweight and obesity girls and boys aged 6–18 years (criteria of IOTF).

BMI categories	Sex				
	Boys *n (%)*	PR (95% CI)	Girls *n (%)*	PR (95% CI)	*p*
Underweight	711 (10.8)	(REF)	1377 (19.3)	1.788 (1.644–2.945)	<0.001
Normal	4971 (75.4)	(REF)	5027 (70.4)	0.934 (0.915–0.953)	<0.001
Overweight	761 (11.5)	(REF)	610 (8.5)	0.740 (0.669–0.819)	<0.001
Obesity	153 (2.3)	(REF)	129 (1.8)	0.779 (0.617–0.982)	0.0339
Overweight and obesity	914 (13.9)	(REF)	739 (10.3)	0.747 (0.682–0.818)	<0.001

PR – prevalence ratio, REF – reference.

The values of z-score were determined for the tested children. The specificities of BMI distribution for a particular age (with accuracy to a month) and sex were taken into account during the calculation. Z-scores values were constructed based on the Box-Cox transformation in such a way that 0 corresponds to the median of the standard distribution of BMI for age and gender. Thanks to such standardization, the z-score values can be compared for different age groups and for different sexes. Box-Cox transformation parameters published by the US Centers for Disease Control and Prevention (CDC) were used for calculation. Table 4 lists the average and median values of z-scores in each age group. In a population equivalent to the model, the value of the average or median should be close to 0. In the study group, the average z-scores were clearly greater than 0 (especially for boys) in the younger age groups. This indicates a tendency towards overweight in younger age groups, especially among boys. We recommend the presented values of the median z-score, due to the presence of some divergent observations in some age groups.

**Table 4:** The values of z-scores in every age group.

Age [years]	Z-score BMI			
	Sex			
	Boys		Girls	
	$\bar{x}$	Me	$\bar{x}$	Me
6	0.25	0.47	0.08	0.33
7	0.17	0.28	−0.05	0.04
8	0.16	0.34	−0.10	0.08
9	0.19	0.29	−0.16	−0.04
10	0.13	0.26	−0.19	−0.13
11	−0.11	0.00	−0.34	−0.26
12	−0.17	−0.09	−0.42	−0.35
13	−0.16	−0.04	−0.39	−0.32
14	−0.23	−0.10	−0.36	−0.25
15	−0.21	−0.11	−0.36	−0.29
16	−0.14	−0.11	−0.36	−0.29
17	−0.26	−0.20	−0.37	−0.34
18	−0.04	0.03	−0.28	−0.34

## Discussion

This study is the first to assess the prevalence of underweight, overweight and obesity in children and adolescents from Ukraine. Based on data collected from a representative sample of the population of children in Ukraine, we found that 11.5% of boys and 8.5% of girls aged 6–18 were overweight, 2.3% and 1.8% were obese (criteria of IOTF).

In the twenty-first century, overweight and obesity among children has become a significant problem mainly in developed countries^10,11^. Studies have shown that an excessively high BMI in childhood increases the risk of obesity in adulthood^12^. About 30% of children in Europe are overweight, and about one quarter of those are obese^13^. Obesity is no longer a phenomenon restricted to affluent parts of the world, but is occurring more and more frequently in the European countries undergoing transformation^4^. Overweight and obesity are characteristic for countries of Western Europe, but research shows that the countries of eastern Europe and Asia are beginning to record an increase in the prevalence of overweight and obesity in children^14^. Modernization and economic growth may contribute to an improved standard of living and provide better access to services, but may also cause deterioration of eating habits with a corresponding increase in the risk of diet-related diseases^4^. According to many research studieds, individual risk factors with associations to overweight and obesity include lack of physical activities, prolonged TV watching and playing computer games, frequent consumption of fast food and calorie dense food items. Family level risk factors include higher socioeconomic status and family history of obesity. Increases in childhood obesity foreshadows serious health consequences e.g., early risk for adult morbidity and mortality, premature death, type 2 diabetes, hypertension and lipidemia, cardiovascular disease, asthma and sleep apnoea, lower self-esteem, and psychological and social stress^15–17^.

In Ukraine, 10% of children are overweight and 2.1% are obese (IOTF criteria). The Global Burden of Disease Study based on an examination of participants indicated that in Ukraine in 2013, the ratio of obesity among girls under the age of 20 was 6.5%, and in boys 7.3%^18^. Kalabiska *et al*. observed that ten percent of the Ukrainian boys and about 2% of the Ukrainian girls were classified overweight according to Cole’s age appropriate cut off point. One percent of boys and below 1% of girls were obese^19^. Mazur *et al*., in a ten-year study (1998–2008), reported a secular trend of overweight and obesity in south-eastern Poland in children with a mean age of 10.5 years^20^. This study also indicated a decreasing frequency of obesity in girls and a statistically significant increase in the incidence of overweight in boys, which shows that the trend is dependent on sex within an age group^20^. Similarly, Meigen *et al*. examined the secular trend among German children and observed a significant increase in childhood obesity between 1999 and 2006, which was more pronounced in boys compared to girls^21^.

The prevalence of overweight and obesity in children and adolescents is different in various European countries. The lowest rates of prevalence in both sexes were observed in Slovakia (9%), Turkey and the Netherlands (10.5%). About 14% of obese and overweight children were found in the Czech Republic, Denmark and Germany. In Russia, Greece and Crete, the prevalence ranges from 22% to 24%, while the highest rates were registered in Italy (26%) and the UK (29%)^22^. On the other hand, Whelton *et al*. conducted a survey among 19,617 children and adolescents between the ages of 4 and 16 in Ireland and Northern Ireland and determined overweight and obesity according to standard criteria of IOTF. They observed that the prevalence of overweight and obesity was higher in women than in men in both countries^23^. Almost a quarter of boys (23% in Ireland and Northern Ireland) and more than one quarter of girls (28% in Ireland and 25% in Northern Ireland) were overweight (including obese). In Ireland and Northern Ireland 7% of girls are obese, whereas 5% and 6% of boys are obese in Ireland and Northern Ireland respectively. In Ireland, the highest prevalence of overweight was in 13-year-old girls (32%) and obesity among 7-year-olds was 11%. In Northern Ireland, the highest prevalence of overweight and obesity was observed in 11 and 8 year-old girls at 33% and 13% respectively^23^. The prevalence of overweight and obesity among children is greater in Romania than in Ukraine. In Romania 16.8% of boys and 16.3% of girls were overweight with an additional 7.8% of boys and 6.4% of girls classified as obese. The prevalence of overweight and obesity was greater in boys than in girls^24^. A similar observation was found in Cyprus^11^ and in Brazil^25^. In Cyprus the overall prevalence of obesity was higher in 2010 (8.1%) compared to 2000 (5.9%) and increased at a greater rate in school-aged boys. The overall prevalence of overweight was also higher in 2010 (20.1%) compared to 2000 (16.5%)^11^.

Koirala *et al*. examined 986 children aged 6–13 from Nepal. The analysis showed that 14.6% were overweight and 11.3% were obese^26^. Underweight was found in 10.4% of the respondents^26^. Studies conducted in Delhi, India by Sharma *et al*. have shown that the prevalence of overweight and obesity amounted to 22% and 6.4%^27^. Obesity and overweight among children and adolescents is a growing problem also in South Asia^15^. Both Ukraine and the above-mentioned countries are among the developing countries according to the International Monetary Fund, IMF (World Economic Outlook Database-WEO Groups and Aggregates Information October 2009).

In Serbia, Rakic *et al*. studied a group of more than 12 thousand students aged 15–18 years and found that the prevalence of obesity was nearly 10%, and obesity 5%, and similar results were found in both sexes^22^. The authors also draw attention to malnutrition, which affected 15% of the students^22^. The prevalence of overweight and obesity in north-western Russia, determined according to IOTF criteria among children aged 14–17 years, amounted to 2.3%, and the rate of underweight was 2.3%^28^.

The dependency between socioeconomic status and obesity is also noteworthy. Shrewsbury and Wardle observed that socio-economic status and obesity in children are correlated^29^. Another study has shown that the dependency between socio-economic status and obesity depends on population, gender and age^30^. In Ethiopia the prevalence of overweight and obesity were 14.7 and 5.8%, respectively^31^. Girls (16.5%) were more overweight than boys (12.3%), nevertheless males (8.6%) were more obese than female children (3.8%). In this study, the authors drew attention to the type of school and the occurrence of overweight and obesity. Children attending government schools were 9.2% overweight and in private schools 29.3% were overweight. Similarly, 1.8% of government and 16.3% of private schools children had childhood obesity^31^. In industrialized countries, groups of low socio-economic status are more likely to be obese than their counterparts in countries with high status, so the risk of obesity in developing countries in particular is increased^32^. Recently several industrialized countries reported a stabilization or even a decrease in childhood overweight and obesity prevalence rates^33–36^.

In recent years, more attention in research was devoted to overweight and obesity, and the issue of underweight was less popular. In our study, 15.2% (IOTF criteria) of children were characterized as underweight. In the age group of 12–15 year-old boys, the percentage of subjects underweight was greater than those overweight and obese. The same trend was found in women aged 9–18 years. Roszko-Kirpsza *et al*. report that in the Podlaskie province of Poland, 24.2% of the children studied were observed to be underweight (22.2% of boys and 26.1% of girls), and overweight and obesity was found in 12.5% of children (13.3% of boys and 11.7% of girls)^37^. Studies by Chabros *et al*. among Polish youth aged 11–15 years showed that the prevalence of body weight deficit (determined according to the criterion of Cole^38^) in girls was higher than in boys^39^. The highest percentage of underweight (8.7%) was found in boys aged 14 and in 13-year-old girls (19.3%)^39^. Kolarzyk *et al*. in a study of preschoolers found underweight in 10.4% of children, correct proportions of weight to height in 83.2%, and excessive body weight in 6.4%^40^.

Consumption of highly processed foods and sedentary lifestyles, along with a decrease in physical activity, may contribute to the increased prevalence of overweight and obesity in children and adolescents. On the other hand, some countries continue to have high rates of malnutrition among children which creates a double burden for public health. In many developing countries, overweight and obesity tend to be perceived as a sign of the wealth of the family^4^.

Over the past few decades, in most developing countries whose populations have experienced improvement in socio-economic status, better health is related to access to health care. On the other hand, an improvement of socio-economic status in low- and middle-income countries could mean the possibility of mechanization leading to less intensive daily activity, and an increase in access to highly processed foods and fast food^11,22^. The implementation of a correct dietary policy among small children would allow avoiding nutritional deficiencies and body weight gain due to an unbalanced diet. A number of genetic, behavioral, and environmental factors may account for the increase in the prevalence of overweight and obesity. Economic transformations in Ukraine could also lead to many positive and negative changes in nutrition, physical activity and lifestyle. In addition, numerous studies have shown that obesity is correlated with the socio-economic condition, ethnic background, as well as lifestyle^22,41–44^.

It should also be noted that after the economic transformation of the late 1980s and early 1990s, there was a sharp increase in overweight and obesity in many countries of Eastern Europe.

Ulijaszek and Koziel observed a trend of declining physical activity and differences in wealth in countries of Eastern Europe^45^. They also suggested that those two trends may be related to each other, as more wealth is usually associated with a higher energy intake^45^. In addition, improved financial situations within a population may contribute to a decline in physical activity, through the purchase of cars, televisions or computers^46,47^. Obesity in childhood carries an increased risk of maintaining this condition in adulthood. Research indicates the need to take preventive measures in the treatment of overweight and obesity already present in preschool children and to identify risk factors for its formation^10^. The main tasks of prevention should be to develop a health education program and by supporting organizations promoting health in schools and educational institutions.

The research presented herein is the first on such a scale in Ukraine and shows that attention should be paid to the problem of obesity, overweight and underweight of children in Ukraine. The results can be a valuable source of information for the Ukrainian authorities, pediatricians and parents. Special attention is paid to the problem of obesity, overweight and underweight among children aged 6–18 in Eastern Europe.

## Methods

### Participants

The study was conducted in randomly selected primary, secondary and high schools of Ukraine. Sample size was determined with the help of the EPI INFO (StatCalc) software. Assuming a 20% prevalence of overweight in Ukraine^18^ we estimated that the sample should include 6,414 children, with a confidence level of 95% and 1% margin of error. This sample size was increased to minimize possible losses. A multistage random cluster sampling method was used to select the participants aged 6–18 years. Approximately 25,000 children were selected from 50 elementary, secondary and high schools in 20 districts of Ukraine. All students from the selected schools were invited to participate in the study, and 15,456 parental approvals were received for participation of their children in the study. Inclusion criteria were: obtaining informed consent from each participant and their parents or guardians, enrolling in the selected schools, a functional state that allow for self-maintenance of a standing position, not taking medication affecting body weight, and ages between 6 and 18 years old.

Out of 15,456 students, whose parents gave approvals for examination, 1,717 students were excluded from the study for the following reasons: a functional state that does not allow for self-maintenance of a standing position (n = 38), taking medication affecting body mass (n = 64), an age less than 6 years or greater than 18 years (n = 98), a lack of desire to participate in the study or a strong pre-test anxiety (n = 52), and being absent from in school on assessment days (n = 1,465). Ultimately, the study group consisted of 13,739 children and adolescents aged 6.0–18.9 years.

Among the representative sample of 13,739 students who were included in this study, 7,143 (52%) were females and 6,596 (48%) were males.

### Anthropometrics measurements

The study took place at nursing clinics. In order for a test procedure to be reliable and reproducible, examinations were carried out at the same time of a day during morning hours, using the same test equipment. The assessments were performed by researchers that have extensive experience.

For each participant, height and weight were measured. These measurements were made in compliance with WHO recommendations, with the students in their underwear and without shoes. Each measurement was taken as the mean of three consecutive measurements.

### Body height and body mass

In children, measurements of body weight and height were performed in triplicate. Body weight was measured with the medical scale RADWAG WPT 60/150 (RADWAG) with the accuracy of 100 g, and height of the children with a stadiometer attached to the scales with the accuracy of 0.1 cm. All the scales were tared using a standardized 1 kg weight.

Mean values of height and weight were obtained from three measurements in order to calculate BMI according to the formula: BMI = body weight in kg/height in m^2^. A BMI-z-score was also calculated.

Three criteria were used in our study to define childhood underweight, overweight and obesity: The International Obesity Task Force (IOTF) reference^48^. The IOTF provided sex- and age-specific BMI cut-off points that correspond to the BMI cut-off points used in defining underweight, overweight and obesity in adults, 18.5, 25 and 30, respectively. World Health Organization (WHO) child growth standard: based on the WHO Reference 2007^49^, children aged 6–19 years are overweight and obese with excess weight over 1 SD and 2 SD, respectively and underweight under 2 SD. The US Center for Disease Control and Prevention (CDC) 2000 Growth Charts: The CDC recommends use of the sex- and age-specific 5^th^, 85th and 95th BMI percentile to define underweight, overweight and obesity, respectively^50^.

### Data analysis

Statistical analysis of the results was performed with Statistica 10.1 software. The selection of schools where children and adolescents were screened was performed using the so-called drawing without repetitions function of the Statistica program. Prevalence ratios were calculated with a 95% confidence interval. The z-scores for the studied children were determined. During calculations, BMI distributions for particular age groups (with accuracy to a month) and sex were taken into account. The z-scores were constructed based on the Box-Cox transformation in such a way that the value of 0 corresponds to the median of the standard BMI distribution for a given age and sex. Statistical analysis was performed using STATISTICA 10.0 and EXCEL 2010^51^.

### Ethical Standards Disclosure

This study was conducted according to the guidelines laid down in the Declaration of Helsinki and all procedures involving human subjects were approved by the Bioethics Committee of Rzeszow University, Poland. Written informed consent was obtained from all subjects.
